# Supplementary material for: An Investigation on the Thermally Induced Compatibilization of SBR and α-Methylstyrene/Styrene Resin
Source: Polymers (Basel). 2021 Apr 13;13(8):1267. doi: 10.3390/polym13081267 (PMC8069804; doi:10.3390/polym13081267)
Supplement: Supplementary file 1 [file polymers-13-01267-s001.pdf]

polymers MDPI

Supplementary Material: Thermal Depolymerization of α-Methylstyrene/Styrene Resins Inducing SBR Crosslinking and Self-Compatibilization

Arnaud Wolf, João Paulo Cosas Fernandes, Chuanyu Yan, Reiner Dieden, Laurent Poorters, Marc Weydert and Pierre Verge

1. Molecular characteristics of poly(αMSt-*co*-St) resin

α-methylstyrene/styrene composition is equal to 45:55 (determined by ^{1}H NMR). M_n = 1820 g/mol, M_w = 2140 g/mol, Đ = 1.14 (determined by GPC, polystyrene standard calibration). Glass transition temperature was measured at 65.1 °C (determined by G″ peak in rheological measurements at 1 Hz, cooling ramp 5°C/min).

2. Summary of the poly(αMSt-*co*-St) resins blended in SBR

Table S1. Summary of the poly(αMSt-*co*-St) resins blended in SBR.

Sample reference	m_{resin} (g)	m_{SBR} (g)	Phr concentration
SBR/poly(αMSt-*a*-St)$_{25}$	0.0146	0.0532	**27**
SBR/poly(αMSt-*a*-St)$_{35}$	0.0187	0.0543	**34**
SBR/poly(αMSt-*a*-St)$_{40}$	0.0241	0.0585	**41**
SBR/poly(αMSt-*a*-St)$_{60}$	0.0307	0.0502	**61**
SBR/poly(αMSt-*a*-St)$_{80}$	0.0378	0.0466	**81**
SBR/poly(αMSt-*a*-St)$_{100}$	0.0452	0.0452	**100**
SBR/poly(αMSt-*a*-St)$_{150}$	0.0575	0.0383	**150**

3. Evidence of the irreversibility of resin compatibilization in SBR

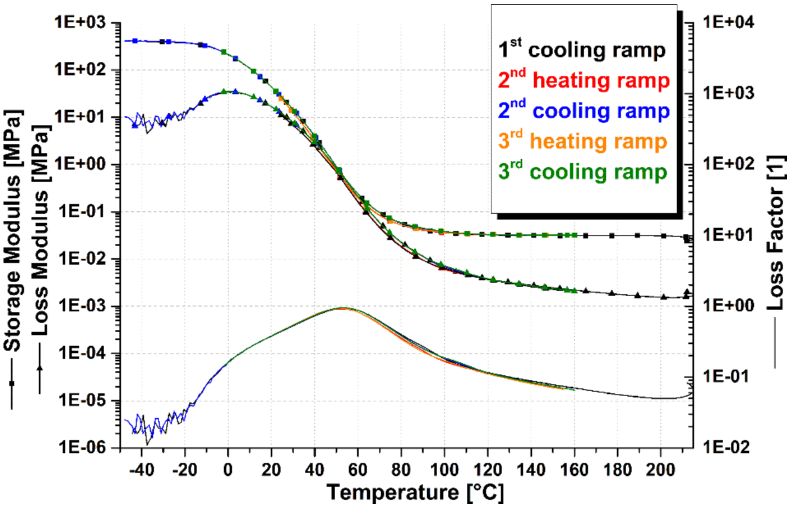

Figure S1. Evidence of the irreversibility of resin compatibilization in SBR after the heat treatment at 215 °C. Identical viscoelastic properties are retained after multiple heat–cool–heat measurements.

4. AFM measurements

Conditions for the sample preparations and AFM measurements explained in the main text Section 2.5.

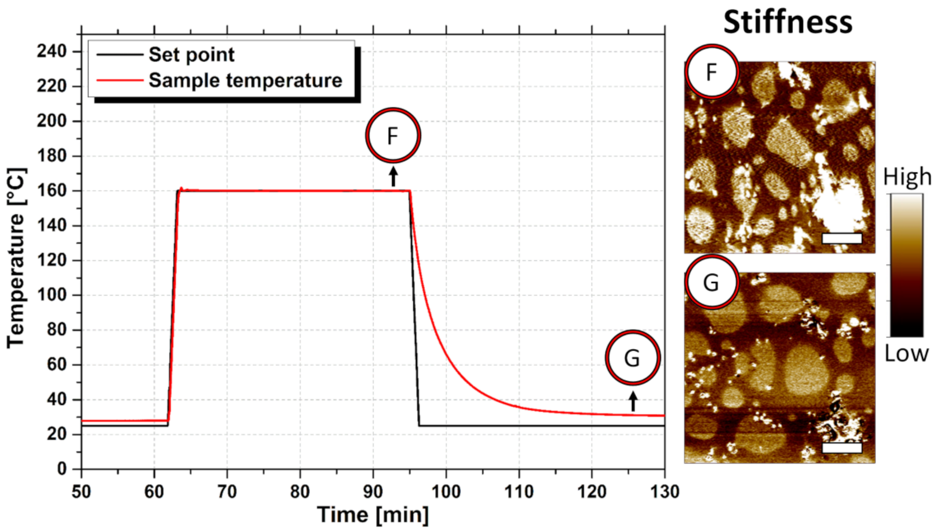

Figure S2. Evidence of the resin/rubber phase separation at 160 °C and after cooling back the sample at 25 °C. Scale bars on the images correspond to 2 μm.

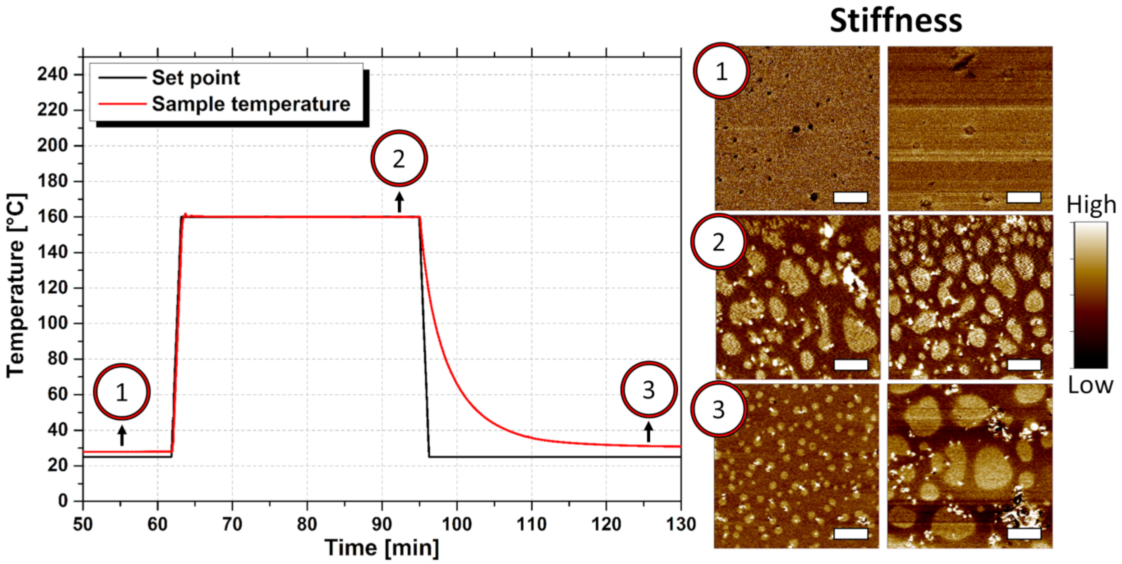

Figure S3. Supplemental images taken at each temperature. Evidences of the phase separation in other sample location. Scale bars on the images correspond to 2 μm.

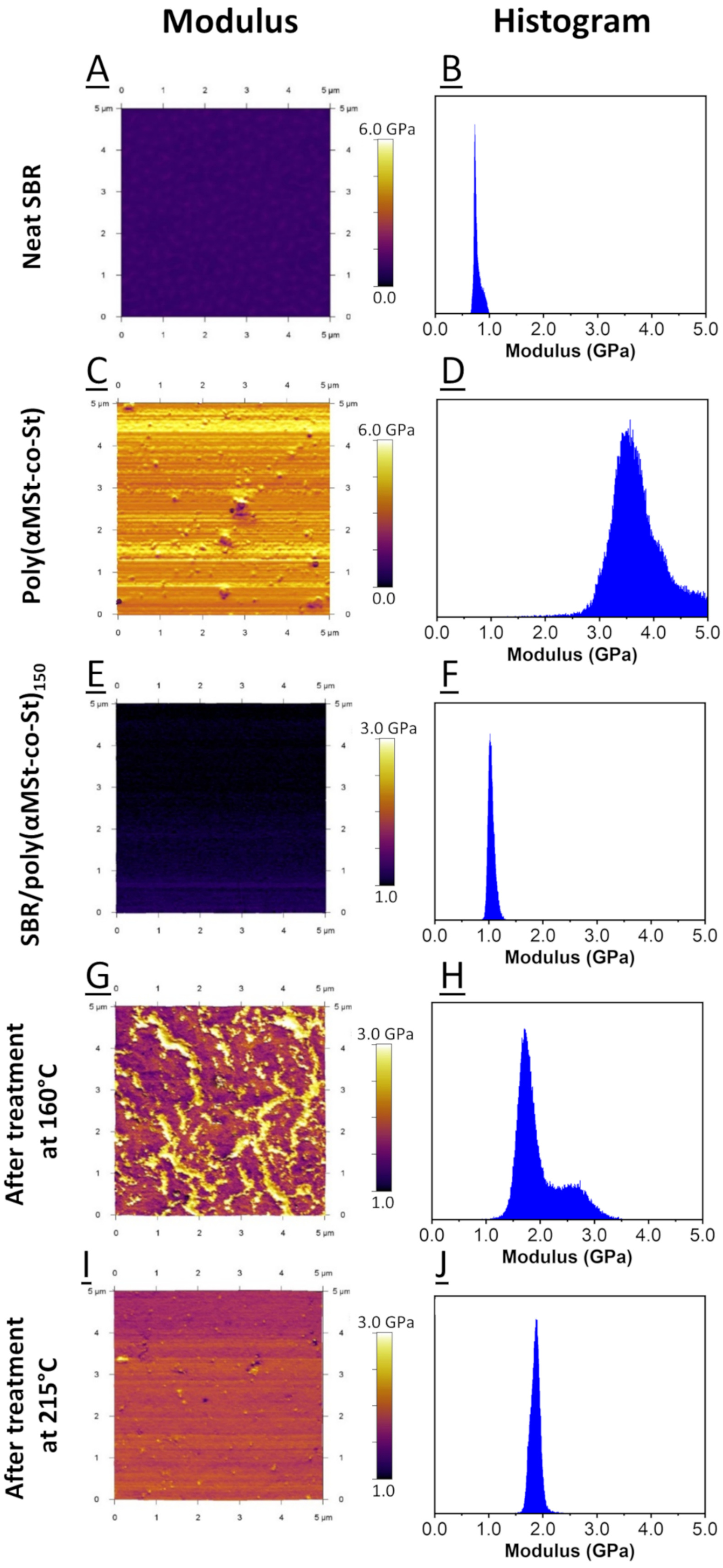

Figure S4. Nanomechanical properties of the different samples.

5. SBR thermal stability

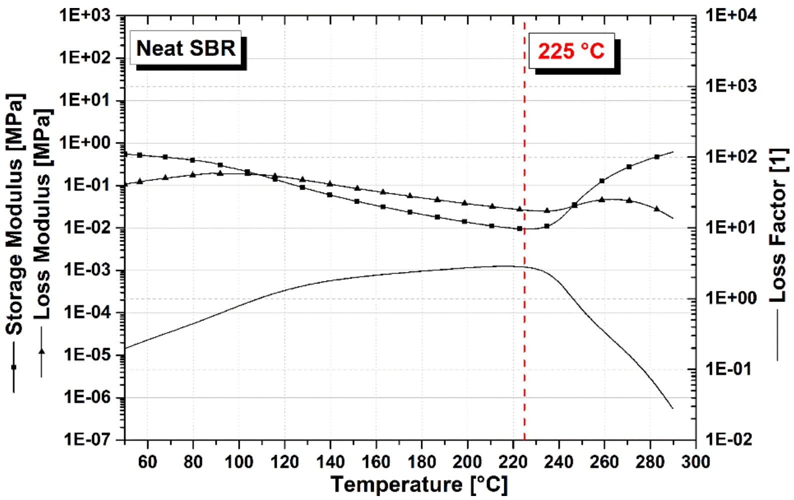

Figure S5. Thermal stability of neat SBR up to 225 °C.

6. NMR experiments

The liquid- and solid-state NMR spectra were measured on a Bruker Avance III HD 600 MHz (proton Larmor frequency) spectrometer. All the chemical shifts were referenced to tetramethylsilane (TMS) by referencing the residual d-chloroform signal to 7.26 ppm (liquid state) or setting the adamantane methylene signal to 37.77 ppm (solid state). For liquid-state NMR, ~ 20 mg of neat SBR was dissolved in ~600 µL of deuterated toluene-d8 (99.6%D, Sigma Aldrich) in a 5 mm NMR tube. ^{13}C spectra with inverse gated ^{1}H decoupling were performed on solution samples with a repetition delay of 6 s and a total accumulation of 10,240 scans. Prior to the swollen solid-state NMR experiments, samples were swollen and washed at least 3 times in 100 mL of toluene. To obtain the solid-state ^{13}C NMR spectra, ~ 7 mg of crosslinked SBR (either with the resin CrR-SBR or with dicumyl peroxide CrDCP-SBR) was swollen by ~ 24 mg of toluene-d8 in a disposable HRMAS insert (B4493, Bruker), which was inserted into a 4 mm ZrO_2 rotor and spun at 7 kHz spinning rate on a double-resonance MAS probe. One-dimensional ^{13}C direct polarization with high-power decoupling NMR experiments were performed on the CrR-SBR and CrDCP-SBR samples with a repetition delay of 4 s, and a total accumulation of 3288 and 2499 scans, respectively.

7. Radical SBR crosslinking with dicumyl peroxide (CrDCP-SBR)

The SBR blend was prepared with 0.1 phr of dicumyl peroxide (DCP). SBR was crosslinked in an oven at 160 °C during 20 min. The crosslinking density of CrDCP-SBR was measured by swelling test (V_e = 1.6x10^{-3}).

8. Viscoelastic behavior of resin-crosslinked SBR (CrR-SBR)

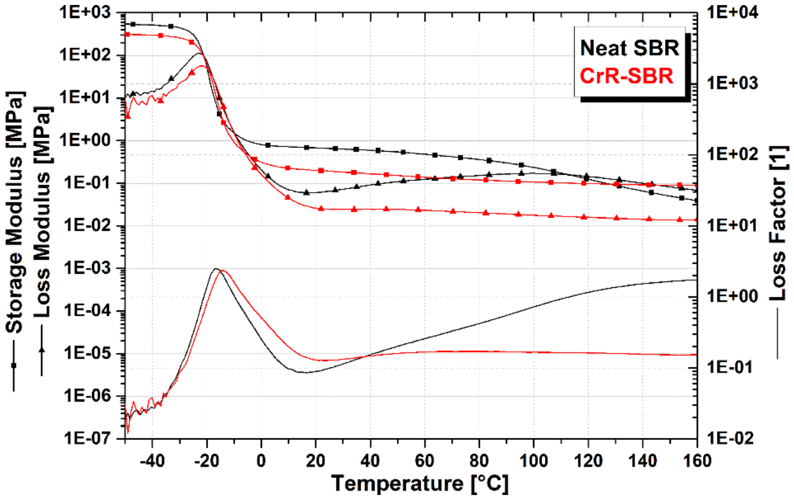

Figure S6. Comparison of the viscoelastic behavior of neat SBR versus CrR-SBR.
